# Patterns of Prefrontal Activation and Performance During Walking Tasks Among Older Adults

**DOI:** 10.1093/geroni/igaa057.2869

**Published:** 2020-12-16

**Authors:** Nemin Chen, Theodore Huppert, Robert Krafty, Andrea Rosso

**Affiliations:** 1 University of pittsburgh, Pittsburgh, Pennsylvania, United States; 2 University of Pittsburgh, Pittsburgh, Pennsylvania, United States; 3 School of Public Health, University of Pittsburgh, Pittsburgh, Pennsylvania, United States

## Abstract

Differences in prefrontal cortex (PFC) control of walking in older age likely arise from changes in neural capacity and compensation. PFC activation by changes in oxygenated hemoglobin from functional near infra-red spectroscopy was examined in 29 older adults (mean age=76). Tasks included standing with cognitive challenge and walking with and without cognitive challenge on even and uneven surfaces. Three PFC activation-performance patterns were identified using K-means clustering: 1) low activation during walking tasks and high activation during standing cognitive task, with the best performance in terms of walking speed and cognitive performance (n=10); 2) low activation on all tasks, with the lowest performance (n=15); 3) high activation during walking and low activation during cognitive, with intermediate performance (n=5). Associations of patterns with cognitive function and structural neuroimaging were explored, with results informing interpretation of functional changes of PFC during aging process, including compensatory mechanisms for primary network impairment.

